# Multi-Vessel Coronary Artery Ectasia

**DOI:** 10.7759/cureus.16584

**Published:** 2021-07-23

**Authors:** Muhammad Madkour, Patrick Hu

**Affiliations:** 1 Internal Medicine, University of California Riverside (UCR) School of Medicine/Riverside Community Hospital, Riverside, USA; 2 Cardiovascular Disease/Interventional Cardiology, University of California Riverside (UCR) School of Medicine/Riverside Community Hospital, Riverside, USA

**Keywords:** coronary artery angiography, anticoagulation, left main coronary artery disease (lmcad), percutaneous coronary intervention, coronary artery ectasia (cae)

## Abstract

Coronary artery ectasia is a relatively rare entity, especially when it involves the left main coronary artery. Furthermore, it is even more uncommon for such a disease process to involve multiple coronary arteries. Here we describe a case of a 78-year-old female who did not possess any of the common risk factors or vasculitic etiologies associated with coronary artery ectasia, who was found to have multi-vessel ectatic segments, including that of the left main coronary artery. This case illuminates the difficult decision making regarding stenting of the coronary arteries with ectatic segments and the decision to anticoagulate.

## Introduction

Coronary artery ectasia (CAE) is defined as focal or diffuse vessel dilatation that exceeds the diameter of the adjacent reference segments by 1.5 times [1]. CAE is well recognized, but a rare finding encountered incidentally on diagnostic coronary angiography with incidences ranging from 1.4 to 4.9% [1]. These ectatic segments may be an isolated finding, or may be in combination with stenotic/obstructive lesions. The right coronary artery (RCA) is the most commonly affected (up to 85% of cases), followed by the left circumflex (LCx) and the left anterior descending (LAD) coronary artery. Left main coronary involvement is extremely rare (0.1% of the population) [2-4]. CAE is classified into four groups [5]:

Type I: Diffuse ectasia of two or more vessels

Type II: Diffuse ectasia in one vessel and localized disease in another vessel

Type III: Diffuse ectasia in one vessel only

Type IV: Localized or segmental involvement

The mechanism is thought to be due to destruction of the vessel media, resulting in increased wall stress and subsequent dilation [2,6]. Other experts suggest a hypothesis of remodeling, where ectatic segments represent an exaggerated form of expansive vascular remodeling in response to plaque growth within the vessel walls [6]. CAE is frequently seen in association with atherosclerotic disease secondary to smoking, hyperlipidemia and uncontrolled hypertension, which comprises nearly half of the reported cases. The rest of the CAE cases have been described secondary to a sequela of connective tissue or vasculitic coronary disorders (i.e., Ehlers-Danlos syndrome, scleroderma, ANCA (antineutrophil cytoplasmic antibodies)-related vasculitidies, syphilitic aortitis and Kawasaki disease). This phenomenon is important because patients with CAE may have worse outcomes than the general population when presenting with signs and symptoms consistent with acute coronary syndromes (ACS). Due to the stagnant non-laminar flow associated with these segments, they typically present a clinical and diagnostic dilemma because they may serve as a nidus for thrombus formation with the possibility of distal embolization or it may lead to dissection or spams. As a result, antiplatelet or anticoagulation therapy has been recommended for larger caliber segments on the basis of an increased risk of thrombosis and myocardial infarction (MI) [7,8]. However, as with any decision to anticoagulate, the benefits of anticoagulation must outweigh the risk of bleeding.

## Case presentation

A 78-year-old Ecuadorian female with history of mild dementia, hypertension, dyslipidemia and prior inferior MI treated initially with balloon angioplasty in South America followed by RCA stent in 2014 presented with worsening angina. Symptoms occurred 30 minutes after Zumba exercises for the last six months and have been associated with shortness of breath. Medications included aspirin 81mg qd (daily), atenolol 50mg qd, hydralazine 50mg three times a day and atorvastatin 40mg qd. There was no history of febrile childhood illness suggestive of Kawasaki disease. She admits to social tobacco use in her teenage years.

**Figure 1:**
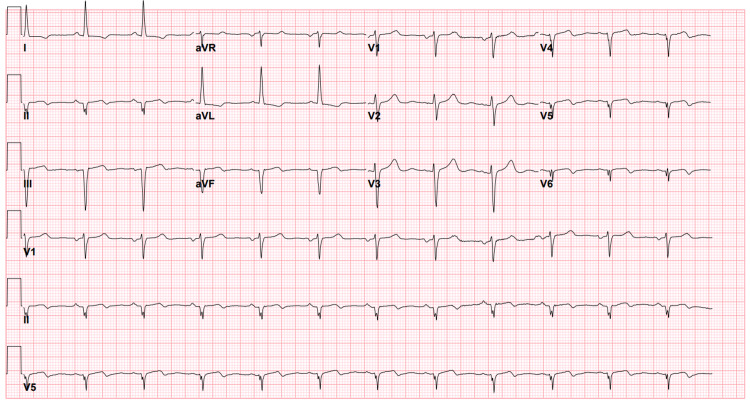
12-lead electrocardiogram shows Q waves in leads II, III, aVF and inverted T waves in leads V4-V6.

On initial assessment, the patient was in no acute distress. Vital signs were stable and oxygen saturation was >94% on room air. Cardiac exam revealed regular heart sounds with no murmur. Pulmonary exam was unremarkable. No jugular venous distension or lower extremity edema was noted. Peak troponin I was elevated at 1.3 ng/ml (normal < 0.08 ng/ml). Electrocardiogram showed Q waves in the inferior leads, inverted T waves in the lateral leads and no ST segment changes indicating acute ischemia (Figure 1). Echocardiogram revealed normal left ventricular function with akinesis in the basal and inferior walls, with an ejection fraction of 60% and no valvular disease.

**Figure 2:**
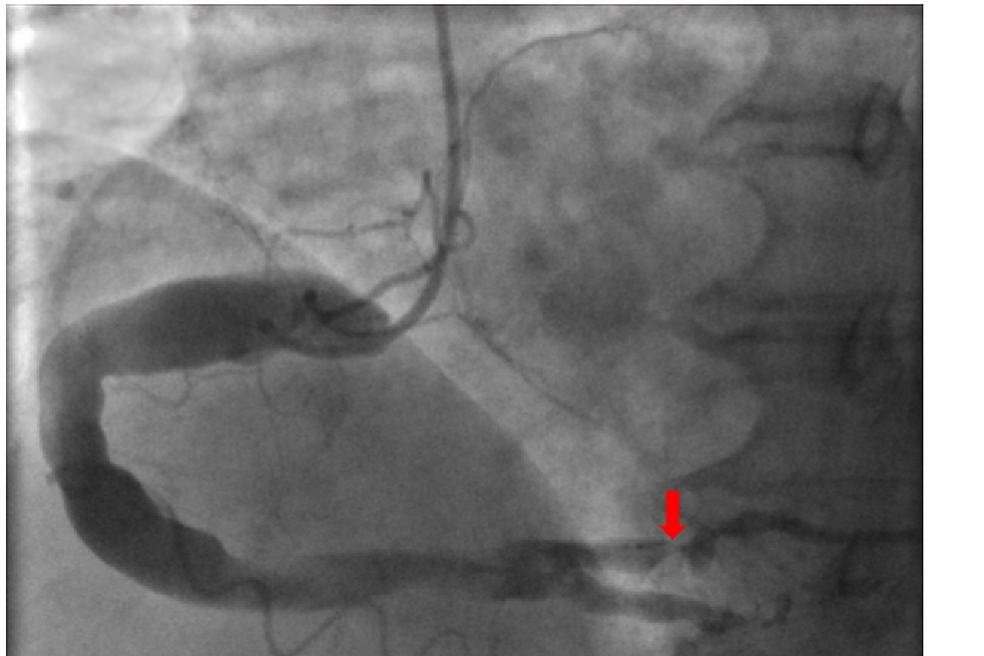
Coronary angiographic image of severely ectatic segments in the proximal and middle right coronary artery. 80-90% de novo stenosis seen in the dominant ostial posterior descending artery (red arrow).

**Figure 3:**
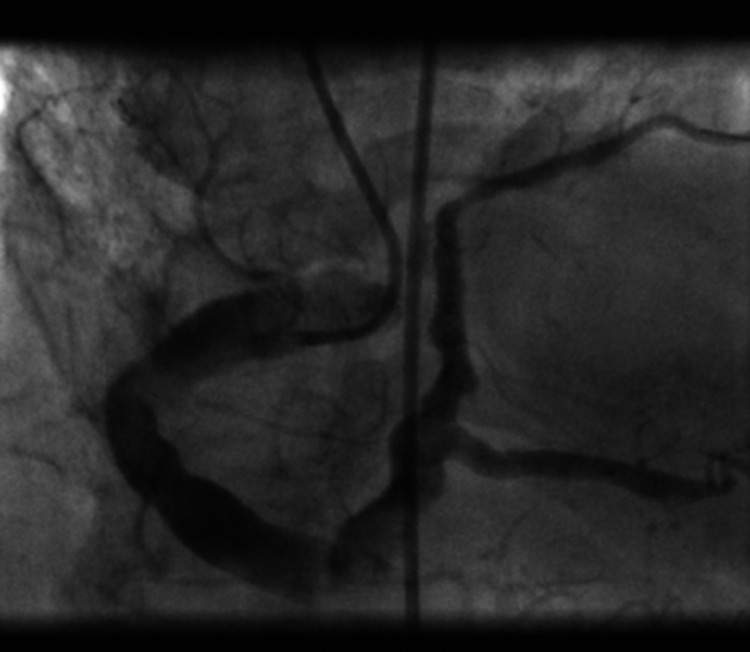
Angiographic image showing excellent flow status post balloon angioplasty and stent placement of the ostial posterior descending artery.

**Figure 4:**
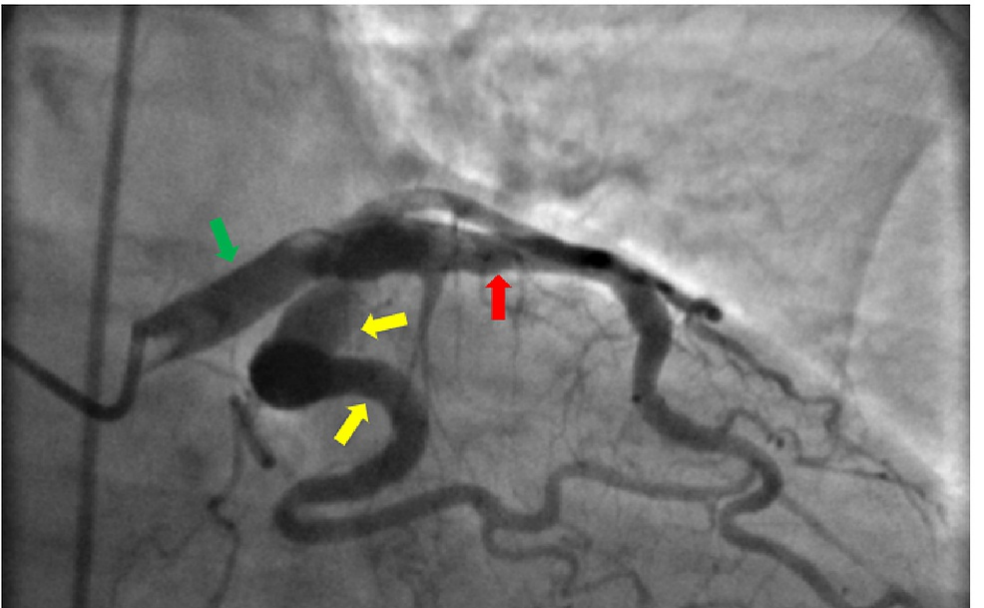
Coronary angiographic image showing severely ectatic segments in the left main coronary artery (green arrow), left anterior descending (LAD) coronary artery (red arrow) and proximal and middle left circumflex coronary artery (yellow arrows).

Coronary angiography showed a dominant RCA that was extremely large in caliber with extreme ectatic segments throughout (Figure 2). 80-90% de novo stenosis in the dominant ostial posterior descending artery (PDA) was noted, with prior stent widely patent in the RCA (Figure 2). Balloon angioplasty was performed with a 2.0 balloon and a drug eluting 4.5 x 12mm Onyx Medtronic stent was deployed to relieve the stenosis. Final angiography showed an excellent flow in both the posterolateral and posterior descending arteries (Figure 3). Furthermore, extreme dilation and severe ectasia was found in the left main coronary artery, LAD, and the left circumflex coronary artery (Figure 4). No significant stenosis was noted in these vessels. No thrombus burden was appreciated. Left ventricular end-diastolic pressure was measured to be 27mmHg with no significant gradient pullback across the aortic valve.

Given the percutaneous coronary intervention (PCI) of the PDA, the patient was initiated on dual anti-platelet therapy (DAPT) with aspirin 81mg and clopidogrel. The patient was initiated on goal-directed medical therapy with high-intensity statin, ace-inhibitor and beta-blocker, and she was safely discharged home without chest pain or any other cardiovascular events with close outpatient follow-up.

## Discussion

Our patient was incidentally found to have extreme dilation and severe ectasia affecting multiple major vessels, and the left main coronary artery was also affected. Given the rarity of this entity, we found it important to shed light on this patient’s unique findings and her management.

Some studies have mentioned that stable angina is the most common presentation of patients with CAE, while others described asymptomatic/incidental finding or denied any specific symptom to the disease [1]. As described earlier, the clinical presentation of a patient with CAE may include symptoms of ACS due to the turbulent blood flow in diseased vessels and thrombus formation. In our case, even though she had initially presented with worsening stable angina, no thrombus was found on coronary angiography, and the culprit lesion in the PDA was stented with PCI. However, her risk of clot burden in the future may increase given the size of the multiple severely ectatic segments.

Treatment for CAE is a controversial topic, as there is lack of clinical trials and standardized guidelines due to the rarity of the disease. Current options include aggressive risk-factor modification (treatment of the underlying atherosclerosis and hypertension), management of the coronary artery disease if obstructive lesions are found, dual anti-platelet therapy (DAPT), antithrombotic therapy if indicated or PCI. Anti-platelet therapy with aspirin has been suggested for all CAE patients since most have coexistent coronary artery obstructive lesions and high likelihood of developing an MI [4,8]. Anticoagulation therapy to prevent coronary thrombus formation has been a debatable topic because of limited randomized trials demonstrating its benefit in CAE. The therapeutic decision should be focused on how to reduce the heavy thrombus burden to improve coronary flow. If the patient’s response to standard DAPT is inadequate, then intensified antithrombotic regimen with warfarin or novel oral anticoagulants (NOACs) may provide an alternative in combination with DAPT in situations of multiple massively ectatic segments in selected high-risk patients.

Upon literature review, outcome data on PCI for incidental CAE is limited to a small subset of case reports and currently there is no evidence-based guideline for stenotic ectatic coronary arteries. PCI of an ectatic culprit vessel in the setting of ACS has been associated with worse outcomes, lower procedural success, higher incidence of no-reflow, higher rates of subsequent stent thrombosis, and long-term mortality [9,10]. These poor outcomes are partially due to the considerable technical challenges with deploying stents in torturous/dilated vessels of different sizes in the coronary tree. Based on our literature search, there have been a few interventional techniques that have been described to attempt to overcome these technical challenges; however, their use is very limited. In patients with proximal disease of the RCA, a covered stent or double open cell stent implantation has been described with good outcomes [11]. On the contrary, in patients with more difficult anatomy with a large side branch originating near an ectatic segment, they may benefit from advanced and complicated PCI techniques such as stent-assisted coiling to conserve the side branch [12]. Nonetheless, even though a few case series have shown some promising success, no standardized interventional guidelines have been formulated for PCI in the setting of incidental CAE.

In our case of Type I ectatic coronary arteries, the decision was made that the patient was not high risk for recurrent thrombosis and stenosis. Given her history of mild-dementia, the decision to anticoagulate was deferred given her potential fall risk. She was placed on aspirin and clopidogrel as a result of the PCI stent. The patient has been symptom free during outpatient follow-up at three and six months post discharge.

Overall, the management of CAE often poses a significant challenge due to the paucity of evidence supporting a specific treatment strategy. The treatment of CAE remains a vexing clinical question and the decision of therapy must take an individualized approach accounting for the patient’s anatomical and clinical risk factors, until further prospective studies are done to evaluate the clinical efficacy of these approaches.

## Conclusions

CAE is an uncommon finding during coronary angiography. We present a case of diffuse CAE involving the left main coronary artery, and highlight the challenges of the interventional and medical management options of these patients. Anticoagulation can be beneficial in select cases, and overall management is guided by the extent of occlusion similar to obstructive coronary artery disease.
